# A plasma proteomic frailty signature for cardiovascular risk stratification: A multi-cohort study

**DOI:** 10.1016/j.ijcha.2026.102035

**Published:** 2026-11-01

**Authors:** Pingjing Zheng, Jiabo Wu, Xiaoqing Gu, Ying Yu, Xinying Fan, Guanghui Wang, Wendi Yan, Gaocheng Gu, Zhangwei Chen, Zhuang Tian, Yang Zhang, Jie Gu, Juying Qian, Junbo Ge

**Affiliations:** a Department of Cardiology, State Key Laboratory of Cardiovascular Diseases, Shanghai Institute of Cardiovascular Diseases, Zhongshan Hospital, Fudan University, China; b NHC Key Laboratory of Ischemic Heart Diseases; c Key Laboratory of Viral Heart Diseases, Chinese Academy of Medical Sciences, China; d National Clinical Research Center for Interventional Medicine, Shanghai, China; e Department of Cardiology, State Key Laboratory of Complex Severe and Rare Diseases, Peking Union Medical College Hospital, Chinese Academy of Medical Sciences & Peking Union Medical College, Beijing 100730, China; f Department of General Practice, Xidu Community Health Center of the Fengxian District, Shanghai, China; g International Medical Center, Zhongshan Hospital, Fudan University, Shanghai, China; h Department of General Practice, Zhongshan Hospital of Fudan University, Shanghai, China

**Keywords:** Frailty, Cardiovascular disease, Risk prediction, Proteomics, Cohort study

## Abstract

**Background:**

Frailty predicts adverse health outcomes, but its relationship with cardiovascular disease (CVD) requires further validation across diverse populations. The value of a circulating protein signature for cardiovascular risk assessment remains uncertain.

**Methods:**

We examined frailty–CVD associations in the XiDu2017 cohort (*n* = 4323) and UK Biobank (UKB; *n* = 84,276) using logistic and Cox regression, respectively. XiDu longitudinal analyses included 844 participants without baseline CVD. In the UKB proteomic subset, a 20-protein frailty (PF) score was compared with clinical frailty and a model of conventional cardiovascular risk factors.

**Results:**

Each higher frailty category was associated with prevalent CVD in XiDu (adjusted odds ratio, 1.120; 95% confidence interval [CI], 1.004–1.251). In UKB, frail participants had a higher CVD hazard than robust participants (adjusted hazard ratio, 1.38; 95% CI, 1.28–1.48). For heart failure, the 5-year area under the curve (AUC) was 0.756 for PF and 0.595 for clinical frailty. Adding PF to conventional risk factors increased the optimism-corrected heart failure AUC by 0.113 (95% CI, 0.077–0.142).

**Conclusions:**

Clinical frailty was associated with cardiovascular outcomes in both cohorts. PF showed incremental discrimination for selected outcomes, particularly heart failure. External validation and evaluation of clinical utility are needed before clinical application.

## 1 Introduction

Frailty reflects reduced physiological reserve and vulnerability to adverse health outcomes [1], [2], [3], including falls, disability, hospitalization, and mortality [4], [5], [6], [7]. Its estimated prevalence among community-dwelling older adults is 10.7% [8]. Frailty and cardiovascular disease (CVD) share age-related vulnerabilities [9] and frequently coexist [10], [11], [12], [13]. Understanding their association may inform cardiovascular care in older populations.

Cross-sectional and longitudinal studies have linked frailty to prevalent and incident CVD [14], [15], [16], [17], with adverse outcomes also reported in pre-frail individuals [18]. Diet, depressive symptoms, and inflammatory processes may contribute to these associations [19], [20], [21], [22], [23]. However, clinical frailty assessments may not fully capture the biological processes underlying cardiovascular vulnerability. Existing cohort studies [24], [25], [26], [27] also differ in population characteristics and outcome assessment, limiting direct comparisons.

A circulating protein signature could provide complementary information. However, a score selected to predict frailty does not necessarily discriminate cardiovascular outcomes, and performance against a clinical frailty measure alone does not establish added value beyond conventional risk factors. Both comparisons are therefore needed. Functional annotation of the selected proteins may also generate hypotheses about shared biological processes.

We used the Chinese XiDu community cohort to examine associations of clinical frailty with prevalent CVD and subsequent CVD or death. UK Biobank (UKB) provided a larger prospective assessment of clinical frailty and incident cardiovascular outcomes. Within the UKB proteomic subset, we evaluated a 20-protein frailty score against clinical frailty and then assessed its incremental discrimination beyond conventional cardiovascular risk factors. Exploratory enrichment analyses characterized the selected proteins. The two clinical cohorts provide complementary evidence on frailty–CVD associations.

## 2 Methods

### 2.1 Study design and population

The XiDu cohort provided cross-sectional and longitudinal community data, while UKB provided prospective cardiovascular outcomes and plasma proteomic measurements.

#### 2.1.1 XiDu cohort

The XiDu study enrolled 5166 residents of Fengxian, Shanghai, in 2017. Administrative redistricting subsequently restricted the 2024 survey to two original sub-districts. That survey included both returning participants and newly recruited residents. Its characteristics are reported separately ***(Table S1)***. Newly recruited residents did not enter the 2017–2024 longitudinal analysis.

#### 2.1.2 UK biobank

UKB baseline measurements were obtained at the initial assessment visit. Ethics approvals and consent procedures for both cohorts are reported in the Ethics statement.

#### 2.1.3 Participant selection

The XiDu2017 analysis included 4323 participants with complete Fried Frailty Phenotype (FFP) data. Of 2746 participants without baseline CVD, 844 contributed to the longitudinal analysis and 1902 did not. The UKB clinical analysis included 84,276 participants aged ≥65 years without baseline CVD **(**Fig. 1**)**.

**Fig. 1:**
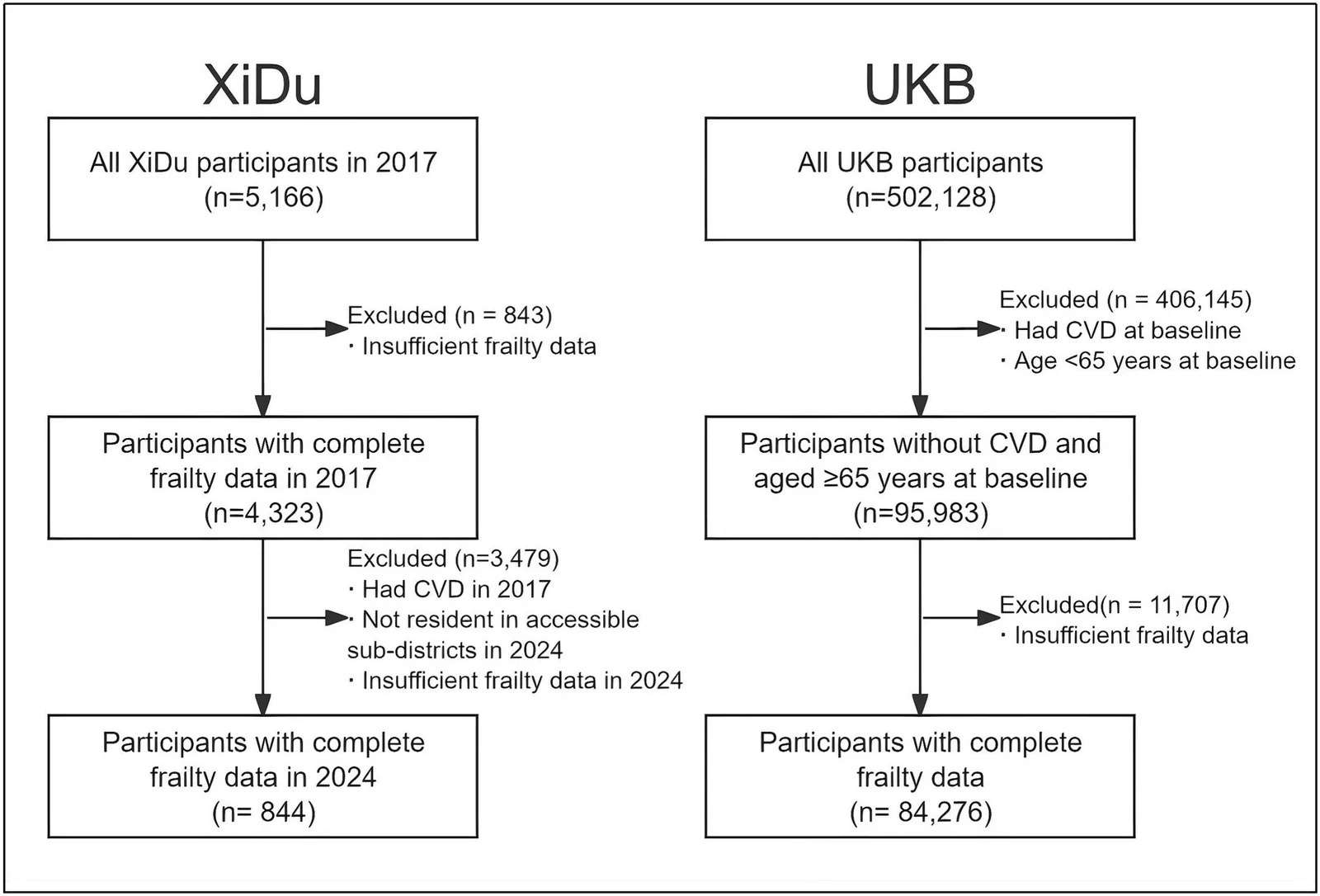
Participant selection. Selection of participants for the XiDu cross-sectional and longitudinal analyses and the UKB clinical analysis. CVD, cardiovascular disease; UKB, UK Biobank.

### 2.2 Assessment of frailty

Frailty was assessed using the five FFP domains: unintentional weight loss, exhaustion, weakness, slowness, and low physical activity [28]. XiDu assessment procedures and International Physical Activity Questionnaire scoring are described in ***Tables S2*** and ***S3***, respectively [29]. UKB used cohort-specific measures of these domains.

Participants were classified as **robust** (0 criteria), **pre-frail** (1–2 criteria), or **frail** (≥3 criteria).

### 2.3 Covariates

Candidate covariates comprised demographic, anthropometric, metabolic, and renal measures available in each cohort. The complete candidate sets and category coding are described in the Supplementary Methods.

### 2.4 Proteomic profiling and preprocessing

UKB plasma proteins were measured using the Olink Explore 3072 platform and supplied as normalized protein expression (NPX) values. Platform-level quality control and normalization have been described previously [30]. Study-specific preprocessing comprised exclusion of proteins with >20% missing measurements, protein-specific median imputation of remaining missing values, and z-score standardization before model fitting. Further details are provided in the Supplementary Methods.

### 2.5 Outcome ascertainment

XiDu prevalent CVD was based on self-reported physician diagnoses of stroke, coronary heart disease (CHD), or other heart disease, which the questionnaire did not further subclassify. The longitudinal endpoint was incident CVD identified at the 2024 survey or all-cause death during follow-up. Deaths were confirmed using official records or family reports.

UKB outcomes comprised acute myocardial infarction (AMI), CHD, heart failure (HF), atrial fibrillation (AF), stroke, and major adverse cardiovascular events (MACE). Overall CVD was defined as a composite of AMI, CHD, HF, AF, and stroke. Diagnosis codes and linked-record sources are detailed in ***Table S4***. Follow-up ended at the first outcome event, death, or 31 October 2022, whichever occurred first.

### 2.6 Statistical analyses

Categorical and continuous variables are summarized as counts (percentages) and means (standard deviations), respectively. XiDu logistic models evaluated prevalent CVD. The multivariable model entered FFP as an ordinal variable (0, robust; 1, pre-frail; 2, frail); odds ratios therefore represent a one-category increase. All 11 variables meeting the univariable criterion of *P* < 0.20, including FFP, were entered simultaneously. Total cholesterol did not meet this criterion. Variance inflation factors were < 5 (maximum, 1.394).

A sensitivity model adjusted for age, sex, diabetes mellitus (DM), body mass index (BMI), total cholesterol (TC), and triglycerides (TG), irrespective of univariable *P* values. Among XiDu participants without baseline CVD, included and non-included participants were compared using absolute standardized mean differences (SMDs). Longitudinal endpoint proportions were compared across FFP categories using Pearson's chi-square test.

UKB clinical frailty associations were evaluated using Cox regression. The multivariable model used robust participants as the reference category. After univariable analyses, least absolute shrinkage and selection operator (LASSO) selection [31] retained age, smoking history, BMI, waist and hip circumferences, serum creatinine (Scr), low-density lipoprotein cholesterol (LDL-C), and TC as adjustment covariates. The cohort-specific selection procedures and their limitations are described in the Supplementary Methods.

The protein-based frailty (PF) score was a weighted sum of 20 proteins selected by LASSO to predict clinical frailty. Coefficients are provided in ***Table S5***. PF and clinical frailty (CF) were compared using Harrell's C-index and 5-year time-dependent area under the receiver operating characteristic curve (AUC), with 95% confidence intervals (CIs) and between-model comparisons. Exploratory Gene Ontology (GO) and Kyoto Encyclopedia of Genes and Genomes (KEGG) enrichment analyses characterized the selected proteins.

Proportional hazards (PH) were assessed using Schoenfeld residual-based tests for the frailty terms and global models in the clinical FFP analysis and the endpoint-specific adjusted CF/PF analyses ***(Table S6)***. *P* < 0.05 was considered evidence against proportional hazards. To further investigate potential departures from the PH assumption, we performed interval-specific Cox sensitivity analyses that allowed frailty-related hazard ratios to vary across three follow-up periods: <5, 5 to <10, and ≥ 10 years. Adjusted hazard ratios with 95% confidence intervals were estimated for each period, and *P* values for heterogeneity across periods were reported ***(Table S7)***.

Incremental discrimination was assessed by adding PF to a base Cox model containing age, sex, smoking status, systolic blood pressure, DM, TC, and high-density lipoprotein cholesterol (HDL—C). These analyses used 500 bootstrap resamples for optimism correction and uncertainty estimation. Tests were two-sided. Analyses used R 4.1.2; the XiDu fixed-covariate sensitivity model used Python 3.11.15 and statsmodels 0.14.6.

## 3 Results

### 3.1 XiDu baseline characteristics and prevalent CVD

XiDu2017 included 4323 participants (58.5% female; mean age, 70.28 years). Frail participants were older, more often female, and more frequently had DM and CVD than robust participants ***(Table S8)***. Descriptive statistics before imputation are provided in ***Table S9***.

CVD prevalence increased across FFP categories (χ^2^ = 11.10; *P* < 0.01; ***Fig. S1***). Compared with robust participants, the unadjusted odds ratio was 1.207 (95% CI, 1.061–1.373; *P* = 0.004) for pre-frail participants and 1.367 (95% CI, 1.064–1.757; *P* = 0.015) for frail participants ***(Table S10)***. None of the five individual FFP components showed a significant univariable association with prevalent CVD.

After multivariable adjustment, each higher FFP category was associated with higher odds of prevalent CVD (adjusted odds ratio [aOR], 1.120; 95% CI, 1.004–1.251; *P* = 0.043; ***Table S11***).

The fixed-covariate sensitivity model gave a similar estimate (aOR, 1.133; 95% CI, 1.015–1.266; *P* = 0.026; ***Table S12***), indicating a comparable association with a clinically selected adjustment set.

### 3.2 XiDu longitudinal analysis

Of the 2746 eligible participants, 844 (30.7%) entered the longitudinal analysis. Compared with the 1902 not included, they were older (71.02 vs 69.04 years; SMD, 0.279), more often frail (9.0% vs 5.1%; overall FFP SMD, 0.192), and more often had DM (8.9% vs 5.8%; SMD, 0.119). Differences in sex and most other measured characteristics were smaller ***(Table S13)***.

The proportions experiencing incident CVD or all-cause death by 2024 were 69.3%, 76.3%, and 92.1% in the robust, pre-frail, and frail groups, respectively (χ^2^ = 18.105; *P* < 0.001; ***Table S14***; Fig. 2).

**Fig. 2:**
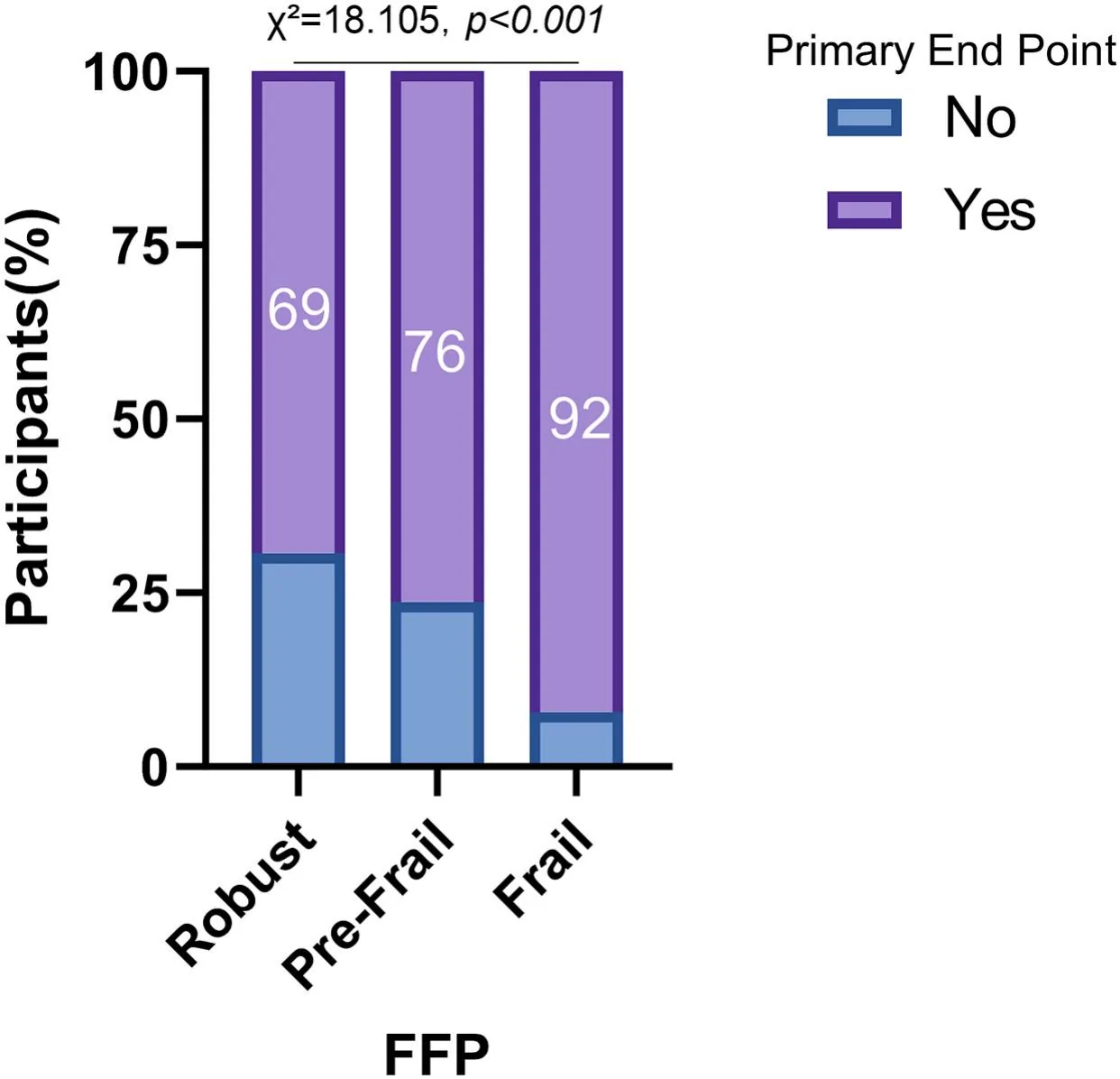
Baseline frailty and the XiDu longitudinal composite endpoint. Proportions experiencing incident CVD or all-cause death by 2024 among 844 participants without baseline CVD, grouped by 2017 frailty category (robust, pre-frail, or frail). Pearson's χ^2^ = 18.105, 2 degrees of freedom; *P* < 0.001. CVD, cardiovascular disease.

### 3.3 Clinical frailty and incident CVD in UKB

Among 84,276 UKB participants, frailty was accompanied by a higher prevalence of DM and other comorbidities and poorer functional status ***(Table S15)***.

In univariable Cox analysis, each additional FFP component was associated with a higher CVD hazard (hazard ratio [HR], 1.09; 95% CI, 1.08–1.11; *P* < 0.001; ***Table S16***).

In the multivariable model, the adjusted HR was 1.38 (95% CI, 1.28–1.48; *P* < 0.001) for frail participants and 1.05 (95% CI, 1.01–1.08; *P* = 0.005) for pre-frail participants, each compared with robust participants ***(Table S17)***.

In interval-specific sensitivity analysis, frail participants had a higher hazard of CVD during the first 5 years (HR, 1.692; 95% CI, 1.561–1.835) and from 5 to <10 years (HR, 1.247; 95% CI, 1.116–1.393). The association was no longer statistically significant at ≥10 years (HR, 0.912; 95% CI, 0.767–1.085; P for heterogeneity <0.001; ***Table S7A***).

### 3.4 Protein frailty score and cardiovascular discrimination

The 20-protein PF signature included proteins related to inflammation (IL1RN) [32], extracellular matrix biology (COL6A3) [33], and growth factor signaling (HGF) [34].

PF discrimination exceeded CF discrimination for several outcomes **(*Table S18*;** Fig. 3**)**. The largest difference was for HF: C-indices were 0.699 (95% CI, 0.677–0.720) and 0.561 (95% CI, 0.541–0.581), and 5-year AUCs were 0.756 (95% CI, 0.723–0.788) and 0.595 (95% CI, 0.563–0.626), respectively (both *P* < 0.001). For MACE, the corresponding C-indices were 0.617 and 0.551 and AUCs were 0.665 and 0.583 (both *P* < 0.001). Both measures also differed significantly for CHD, AF, and stroke. For AMI, the C-index difference was not significant (0.579 vs 0.554; *P* = 0.205), whereas the AUC difference was significant (0.628 vs 0.534; *P* = 0.004). Endpoint-specific HRs are reported in ***Table S19***.

**Fig. 3:**
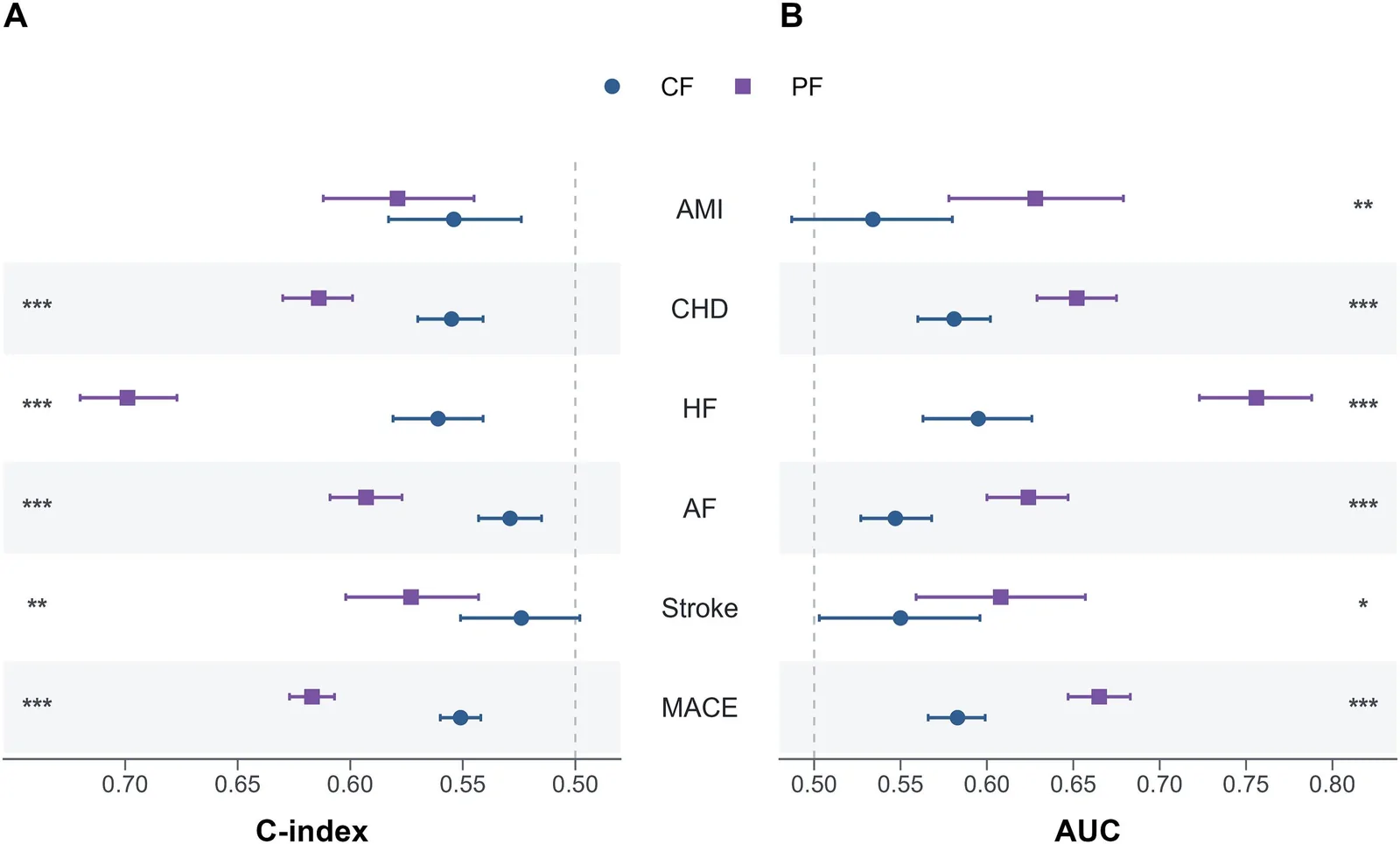
Discrimination of clinical and protein-based frailty for cardiovascular outcomes. (A) Harrell's C-index. (B) Five-year time-dependent AUC. Circles represent CF and squares represent PF; error bars show 95% confidence intervals in the full proteomic cohort. Asterisks denote between-model comparisons: *P < 0.05, **P < 0.01, and ***P < 0.001. CF, clinical frailty; PF, protein-based frailty; AUC, area under the receiver operating characteristic curve; AMI, acute myocardial infarction; CHD, coronary heart disease; HF, heart failure; AF, atrial fibrillation; MACE, major adverse cardiovascular events.

Interval-specific sensitivity analyses in the proteomic subset indicated that associations between frailty measures and cardiovascular outcomes varied over time ***(Table S7B)***. Most notably, the positive association between PF and incident heart failure persisted across all three follow-up periods, although its magnitude attenuated over time. By contrast, the associations of PF with CHD and MACE weakened over longer follow-up, while the direction of the PF–AF effect estimate changed in the ≥10-year period.

Adding PF to conventional risk factors increased the optimism-corrected MACE C-index from 0.603 to 0.643 (ΔC-index, 0.040; 95% CI, 0.033–0.048) and 5-year AUC from 0.631 to 0.690 (ΔAUC, 0.058; 95% CI, 0.045–0.074). For HF, the C-index increased from 0.634 to 0.716 (ΔC-index, 0.082; 95% CI, 0.061–0.101) and 5-year AUC from 0.659 to 0.772 (ΔAUC, 0.113; 95% CI, 0.077–0.142; all *P* < 0.001). Improvements were less consistent for AMI and stroke ***(Table S20)***.

Exploratory enrichment analyses identified GO molecular functions related to growth factor binding, extracellular matrix structure, and fibronectin binding. GO cellular components included endoplasmic reticulum lumen, secretory granule membrane, and collagen trimer. KEGG annotations included extracellular matrix–receptor interaction and focal adhesion **(**Fig. 4**)**. These results provide functional context for the selected panel.

**Fig. 4:**
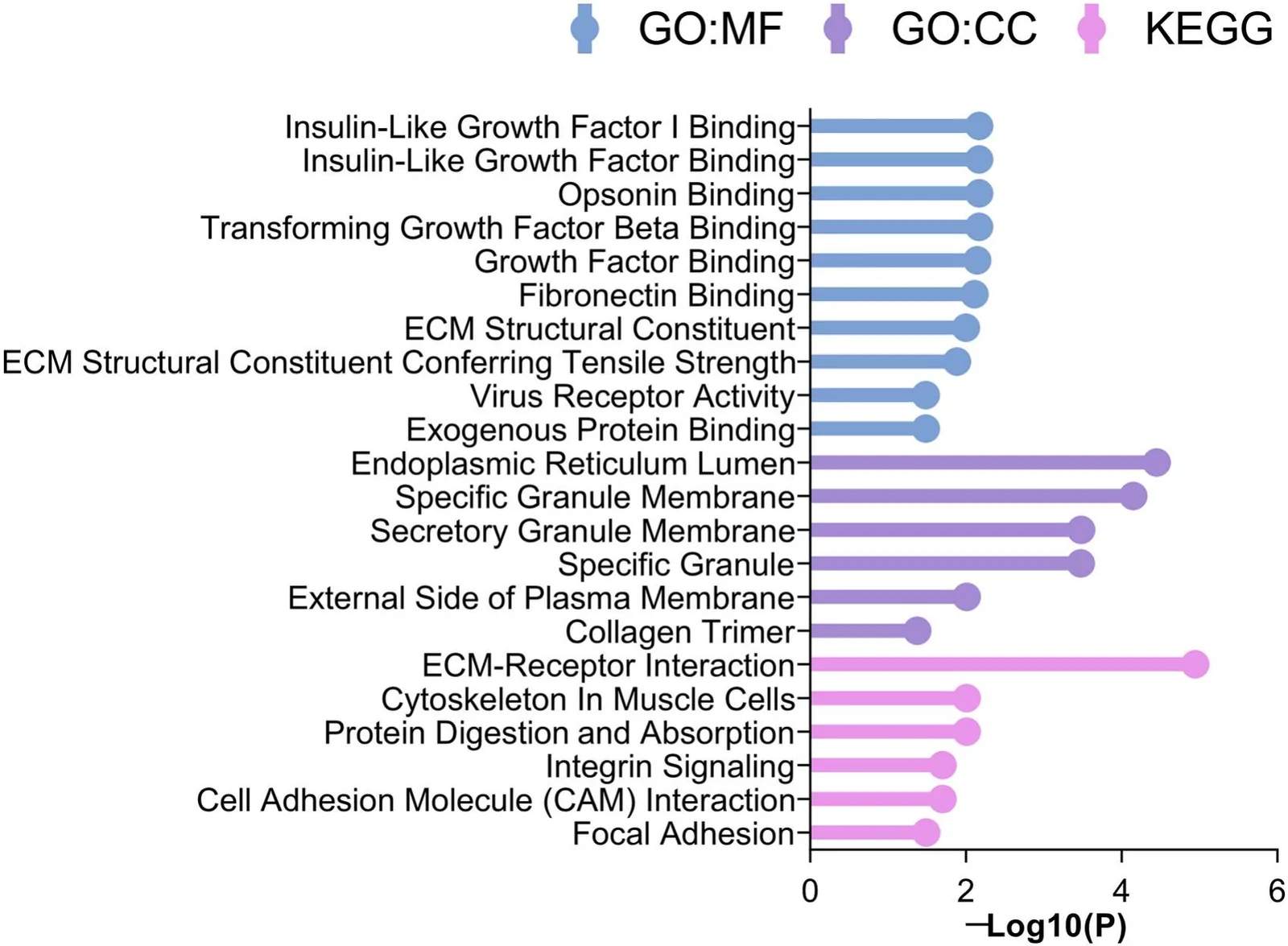
Exploratory functional enrichment of the 20-protein frailty signature. Lollipop plot of functional enrichment for the 20 selected proteins. Blue, Gene Ontology molecular function (GO:MF); purple, GO cellular component (GO:CC); pink, Kyoto Encyclopedia of Genes and Genomes (KEGG) pathways. The horizontal axis shows −log10(P). ECM, extracellular matrix. These analyses provide functional context and do not establish causal mechanisms.

## 4 Discussion

Clinical frailty was associated with prevalent CVD in XiDu and incident CVD in UKB. In the UKB proteomic subset, PF discriminated selected cardiovascular outcomes better than CF and added discrimination beyond conventional risk factors, with the largest gains for HF. These comparisons address distinct questions: PF–CF comparisons assess discrimination relative to a clinical frailty measure, whereas adding PF to the conventional-risk model tests whether the score contains additional cardiovascular risk information. The observed gains support further evaluation of PF as a complementary marker, while the outcome-specific results argue against assuming a uniform benefit across CVD subtypes.

The clinical frailty associations are consistent with earlier studies [17], [18], [20], [35], [36], [37], although differences in outcome ascertainment and frailty measurement preclude treating the two cohorts as direct replications. The inverse TC association in the adjusted UKB model should not be interpreted as evidence of protection. Medication use, residual confounding, and simultaneous inclusion of correlated lipid measures may contribute, but their contributions were not established here. This conditional association does not resolve the broader relationship between lipids and cardiovascular risk [38].

We observed the lack of significant individual association between any single FFP criterion, such as exhaustion, and CVD in the XiDu cohort. Contrasted with the signal from the composite phenotype, it may underscore a concept that frailty is a syndromic risk factor where the whole is greater than the sum of its parts. The clinical implication is that assessment of the integrated phenotype is crucial, rather than focusing on isolated symptoms. Prior proteomic work describes age-related protein variation [39], while experimental studies implicate growth factor signaling and extracellular matrix remodeling in cardiac disease [40], [41], [42]. Circulating proteins have also been associated with late-life HF and frailty [43]. Against this background, enrichment of extracellular matrix and growth factor annotations offers a biological rationale for investigating the larger HF discrimination gains. However, the proteins were selected to predict clinical frailty, and neither enrichment nor discrimination establishes that these pathways mediate the PF–HF association. The finding is a hypothesis for independent biological and prognostic validation.

Although PF improved discrimination for selected cardiovascular outcomes, these statistical gains do not by themselves establish clinical utility, particularly given the modest C-index for MACE (0.617). Further assessment of model calibration and decision-analytic net benefit is needed to determine its potential clinical applicability. We plan targeted assays of selected PF proteins in XiDu as a step toward testing reproducibility in a Chinese community population. External validation of the complete model will still be required.

Several limitations qualify these findings. The observational design cannot establish causation, and residual confounding and reverse causation remain possible. XiDu follow-up included only 30.7% of baseline CVD-free participants, who were older, frailer, and more often diabetic than those not included. This enrichment may raise the observed endpoint frequency, while selection related to both frailty and cardiovascular risk could attenuate or exaggerate the frailty association. Individual reasons for non-inclusion were unavailable, so non-inclusion cannot be equated with loss to follow-up. The direction and magnitude of bias remain uncertain. UKB volunteer selection also limits transportability. Cohort-specific frailty measures, self-reported XiDu diagnoses, and record-based UKB outcomes may produce different misclassification. XiDu additionally combined CVD with all-cause death, limiting direct comparison with UKB cardiovascular endpoints. The PF model was developed and evaluated within the UK Biobank. Although bootstrap-based optimism correction was applied to analyses of incremental discrimination, this internal resampling procedure cannot substitute for independent external validation of the complete proteomic signature. Departures from the PH assumption were detected in several Cox models. In interval-specific sensitivity analyses, several associations varied across intervals. These findings indicate potential time-varying associations and support interpreting the primary HRs as summary estimates over the entire follow-up period. Absolute discrimination was modest for several outcomes, and comparisons across outcomes and enrichment analyses were exploratory. Clinical utility and causal pathways therefore remain unproven.

## 5 Conclusion

Clinical frailty was associated with cardiovascular outcomes in both cohorts, and PF added discrimination for selected UKB outcomes, particularly HF. These findings support further evaluation of frailty-related information in cardiovascular assessment. Whether interventions targeting frailty [44], [45] or use of the PF score improve cardiovascular outcomes was not tested.

## CRediT authorship contribution statement

**Pingjing Zheng:** Writing – original draft. **Jiabo Wu:** Writing – original draft. **Xiaoqing Gu:** Resources, Investigation. **Ying Yu:** Resources, Investigation. **Xinying Fan:** Resources, Investigation. **Guanghui Wang:** Resources, Investigation. **Wendi Yan:** Writing – original draft, Investigation. **Gaocheng Gu:** Investigation. **Zhangwei Chen:** Writing – review & editing, Resources. **Zhuang Tian:** Supervision, Resources. **Yang Zhang:** Writing – review & editing, Resources. **Jie Gu:** Validation, Supervision, Resources, Investigation. **Juying Qian:** Supervision, Resources. **Junbo Ge:** Supervision, Resources.

## Ethics statement

The XiDu study was approved by the Ethics Committee of Zhongshan Hospital, Fudan University (approval No. B2020–077), and the UK Biobank study was approved by the North West Multi-centre Research Ethics Committee. All participants provided written informed consent before enrollment. The study was conducted in accordance with the principles of the Declaration of Helsinki.

## Funding

This work was supported by the 10.13039/501100012166National Key Research and Development Program of China (2023YFC3606500 and 2023YFC2506500); the Special Clinical Research Project of Shanghai Municipal Health Commission (20244Y0022); the Shanghai “Rising Stars of Medical Talent” Youth Development Program [SHWSRS (2024)_070]; the Shanghai Science and Technology Commission (25SF1902100); and the Shanghai Top Priority Research Center Construction Project (2022ZZ01010).

## Declaration of competing interest

The authors declare that they have no known competing financial interests or personal relationships that could have appeared to influence the work reported in this paper.

## Data Availability

XiDu data may be requested from the corresponding authors, subject to ethics approval and an appropriate data-sharing agreement. Access to UKB data is governed by UK Biobank's application and access procedures (https://www.ukbiobank.ac.uk/use-our-data/apply-for-access/; accessed 11 October 2026).
