# Supplementary material for: A plasma proteomic frailty signature for cardiovascular risk stratification: A multi-cohort study
Source: Int J Cardiol Heart Vasc. 2026 Nov 1;67:102035. doi: 10.1016/j.ijcha.2026.102035 (PMC13744895; doi:10.1016/j.ijcha.2026.102035)
Supplement: Supplementary file 1 [file mmc1.docx]

Supplementary data

A Plasma Proteomic Frailty Signature for Cardiovascular Risk Stratification: A Multi-Cohort Study

# Supplementary Methods

## Candidate covariates and model specification

XiDu candidate covariates were age, sex, diabetes mellitus (DM), waist-to-hip ratio (WHR), body mass index (BMI), blood urea nitrogen (BUN), serum creatinine (Scr), hemoglobin (Hb), uric acid (UA), total cholesterol (TC), and triglycerides (TG). DM was coded as present or absent according to the study’s glycemic criteria. The original multivariable logistic model included the Fried Frailty Phenotype (FFP) and all ten non-TC covariates that met P < 0.20 in univariable screening. The fixed-covariate sensitivity analysis retained age, sex, DM, BMI, TC, and TG irrespective of univariable significance.

UK Biobank (UKB) candidate covariates were age, smoking history, alcohol status, DM, BMI, waist circumference, hip circumference, Scr, Hb, high-density and low-density lipoprotein cholesterol (HDL-C and LDL-C), TG, and TC. Smoking history was coded as ever versus never smoking. Alcohol and DM retained the reported UKB response categories. The final clinical frailty Cox model included FFP, age, smoking history, BMI, waist and hip circumferences, Scr, LDL-C, and TC. Cohort-specific availability led to different adjustment strategies; these strategies do not make the two effect estimates directly interchangeable.

## Protein-score construction and discrimination

Proteomic analyses used the normalized protein expression (NPX) measurements released by UKB following platform-level quality control and normalization. Proteins with >20% missing measurements were excluded. For retained proteins, remaining missing values were imputed using the median of each protein. Protein measurements were then z-score standardized using the mean and standard deviation of the model-development data. The protein-based frailty (PF) score was calculated as the sum of the 20 selected protein measurements multiplied by their corresponding coefficients. Comparisons of clinical frailty (CF) with PF use the full proteomic cohort. For incremental discrimination, the conventional-risk model comprised age, sex, smoking status, systolic blood pressure, DM, TC, and HDL-C, and was compared with the same model plus PF. Reported incremental-performance estimates and intervals were derived using 500 bootstrap resamples. Details on the Olink platform, sample quality-control criteria, and normalization procedures are available at: https://www.ukbiobank.ac.uk/analysis/proteomics/olink-proteomics/.

## Table S1. Characteristics of participants in the XiDu2024 survey (n = 1,687)

| **Variable** | **Robust** | **Pre-frail** | **Frail** |
| --- | --- | --- | --- |
| Participants, n (%) | 200 (11.9) | 1262 (74.8) | 225 (13.3) |
| Sex, n (%) |  |  |  |
| Male | 62 (31.0) | 449 (35.6) | 84 (37.3) |
| Female | 138 (69.0) | 813 (64.4) | 141 (62.7) |
| DM, n (%) |  |  |  |
| Present | 19 (9.5) | 166 (13.2) | 33 (14.7) |
| Absent | 181 (90.5) | 1096 (86.8) | 192 (85.3) |
| CVD, n (%) |  |  |  |
| Present | 110 (55.0) | 742 (58.8) | 158 (70.2) |
| Absent | 90 (45.0) | 520 (41.2) | 67 (29.8) |
| Age, years | 73.30 (5.59) | 73.85 (5.42) | 74.26 (5.60) |
| WHR | 0.94 (0.05) | 0.94 (0.05) | 0.94 (0.06) |
| BMI, kg/m² | 24.65 (2.77) | 24.77 (3.26) | 24.84 (3.36) |
| BUN, mmol/L | 6.03 (1.27) | 5.97 (1.50) | 6.07 (1.72) |
| Scr, μmol/L | 64.77 (13.82) | 66.07 (16.16) | 69.24 (20.49) |
| Hb, g/L | 137.70 (9.86) | 139.58 (11.92) | 139.12 (12.51) |
| UA, μmol/L | 305.38 (61.68) | 311.06 (78.03) | 317.95 (81.75) |
| TC, mmol/L | 4.95 (0.84) | 4.95 (0.92) | 5.01 (0.98) |
| TG, mmol/L | 1.67 (0.99) | 1.56 (0.92) | 1.54 (0.88) |
| LDL-C, mmol/L | 2.65 (0.65) | 2.70 (0.71) | 2.71 (0.71) |
| HDL-C, mmol/L | 1.30 (0.25) | 1.32 (0.28) | 1.33 (0.30) |

Values are n (%) or mean (SD). The 2024 survey included both returning participants and newly recruited residents; it is not the 2017–2024 longitudinal analysis sample.

WHR, waist-to-hip ratio; BMI, body mass index; BUN, blood urea nitrogen; Scr, serum creatinine; Hb, hemoglobin; UA, uric acid; TC, total cholesterol; TG, triglycerides; DM, diabetes mellitus; CVD, cardiovascular disease; SD, standard deviation; LDL-C, low-density lipoprotein cholesterol; HDL-C, high-density lipoprotein cholesterol.

## Table S2. Assessment of the Fried Frailty Phenotype in XiDu

| **Criteria** | **Assessment Method** | **Definition** |
| --- | --- | --- |
| Weight Loss | Measured using an electronic scale. | Unintentional weight loss of ≥4.5 kg or ≥5% compared to one year prior. |
| Exhaustion | Asked, "Do you often feel tired?" | An affirmative "Yes" response. |
| Weakness | Measured by dominant hand grip strength using a dynamometer. | <26 kg for men or <18 kg for women. |
| Slowness | Timed over a 4.57-meter walk at usual pace. | Walking speed <0.8 m/s or requiring the use of an assistive device or personal assistance. |
| Low Physical Activity | Assessed using the IPAQ. | Classified as "low" according to the IPAQ scoring protocol. |

FFP categories were robust (0 criteria), pre-frail (1–2 criteria), and frail (≥3 criteria). This table describes the XiDu assessment procedures.

FFP, Fried Frailty Phenotype; IPAQ, International Physical Activity Questionnaire.

## Table S3. Physical activity classification using the IPAQ

| **Step / Category** | **Specific Method / Criteria** |
| --- | --- |
| Data Cleaning and Outlier Handling | 1. Delete data with missing time or frequency. 2. Convert daily cumulative time for each activity to minutes. 3. Exclude participants with missing frequency or duration, and those with a total daily duration of all three intensity activities exceeding 16 hours. 4. Data below 10 minutes per day were recorded as zero. |
| Data Truncation | If the daily duration for a specific intensity activity exceeded 3 hours, it was reset to 3 hours. |
| Calculating Weekly Activity Level | MET value of the activity × weekly frequency (days/week) × daily duration (minutes/day). (Unit: MET-min/week) |
| High Activity Level | Meets either of:   1. Vigorous-intensity activity on ≥3 days achieving ≥1500 MET-min/week 2. Any combination of walking, moderate- or vigorous-intensity activities on ≥7 days achieving ≥3000 MET-min/week. |
| Moderate Activity Level | Meets any of:   1. Vigorous activity ≥20 minutes per day on ≥3 days 2. Moderate-intensity activity and/or walking ≥30 minutes per day on ≥5 days 3. Any combination of activities on ≥5 days achieving ≥600 MET-min/week. |
| Low Activity Level | Meets either of:   1. No reported activity. 2. Reported some activity but does not meet criteria for ‘moderate’ or ‘high’ categories. |

Activity is expressed in MET-min/week. Participants were classified as high, moderate, or low according to the listed criteria.

IPAQ, International Physical Activity Questionnaire; MET, metabolic equivalent of task.

## Table S4. Definitions and ascertainment of UKB cardiovascular outcomes

| **Outcome** | **ICD-10 codes** | **ICD-9 codes** | **UKB fields** | **Data source** |
| --- | --- | --- | --- | --- |
| MACE | I21, I22, I46, I60, I61, I63, I64 | 410, 430, 431, 433, 434, 436 | 41270 (ICD-10), 41271 (ICD-9) | HES |
| AMI | I21, I22 | 410 | 41270 (ICD-10), 41271 (ICD-9) | HES |
| CHD | I20-I25 | 410–414 | 41270 (ICD-10), 41271 (ICD-9) | HES |
| HF | I50 | 428 | 41270 (ICD-10), 41271 (ICD-9) | HES |
| AF | I48 | 427.3 | 41270 (ICD-10), 41271 (ICD-9) | HES |
| Stroke | I60-I64 | 430–434, 436 | 41270 (ICD-10), 41271 (ICD-9) | HES |

Overall CVD was defined as a composite of AMI, CHD, HF, AF, and stroke. Field 41280 contains first inpatient ICD-10 diagnosis dates; the corresponding ICD-9 date field is 41281. Date of death is field 40000. The reported administrative end of follow-up was 31 October 2022.

AMI, acute myocardial infarction; CHD, coronary heart disease; HF, heart failure; AF, atrial fibrillation; MACE, major adverse cardiovascular events; ICD, International Classification of Diseases; HES, Hospital Episode Statistics; UKB, UK Biobank; CVD, cardiovascular disease.

## Table S5. Protein features and coefficients of the PF score

| **Rank** | **Feature** | **Coefficient** |
| --- | --- | --- |
| 1 | ITGAV; Integrin alpha-V | -0.1711 |
| 2 | COL6A3; Collagen alpha-3 (VI) chain | 0.167 |
| 3 | CA14; Carbonic anhydrase 14 | -0.1498 |
| 4 | IL1RN; Interleukin-1 receptor antagonist protein | 0.1116 |
| 5 | SPON2; Spondin-2 | 0.1097 |
| 6 | GAST; Gastrin | 0.0934 |
| 7 | AREG; Amphiregulin | 0.0914 |
| 8 | GFRA1; GDNF family receptor alpha-1 | 0.0816 |
| 9 | HGF; Hepatocyte growth factor | 0.0616 |
| 10 | IL15; Interleukin-15 | 0.0531 |
| 11 | EPO; Erythropoietin | 0.0402 |
| 12 | KRT19; Keratin, type I cytoskeletal 19 | 0.0252 |
| 13 | HAVCR1; Hepatitis A virus cellular receptor 1 | 0.0222 |
| 14 | CCL7; C-C motif chemokine 7 | 0.0215 |
| 15 | PLAUR; Urokinase plasminogen activator surface receptor | 0.0175 |
| 16 | ACHE; Acetylcholinesterase | -0.0165 |
| 17 | ADM; Pro-adrenomedullin | 0.0153 |
| 18 | SERPINA3; Alpha-1-antichymotrypsin | 0.0102 |
| 19 | LTBP2; Latent-transforming growth factor beta-binding protein 2 | 0.0079 |
| 20 | RBFOX3; RNA binding protein fox-1 homolog 3 | 0.0067 |

The PF score is the weighted sum of the selected protein measurements using the coefficients shown.

PF, protein-based frailty.

## Table S6. Schoenfeld residual-based proportional hazards diagnostics in UKB Cox models

### A. UK Biobank clinical FFP Cox model

| **Variable / test** | **df** | **P value** |
| --- | --- | --- |
| FFP (overall 3-category term) | 2 | <0.001 |
| Total cholesterol | 1 | <0.001 |
| LDL cholesterol | 1 | <0.001 |
| Serum creatinine | 1 | <0.001 |
| Hip circumference | 1 | <0.001 |
| Waist circumference | 1 | <0.001 |
| BMI | 1 | <0.001 |
| Ever smoked | 1 | 0.046 |
| Age | 1 | 0.823 |
| GLOBAL | 10 | <0.001 |

FFP was tested jointly across its two non-reference categories (df = 2). The global test evaluates the adjusted model as a whole.

### B. Endpoint-specific adjusted CF and PF Cox models

| **Outcome** | **Events** | **CF term P** | **CF global P** | **PF term P** | **PF global P** |
| --- | --- | --- | --- | --- | --- |
| AMI | 347 | 0.374 | 0.838 | 0.260 | 0.888 |
| CHD | 1413 | <0.001 | <0.001 | <0.001 | <0.001 |
| HF | 745 | <0.001 | 0.009 | <0.001 | <0.001 |
| AF | 1493 | 0.008 | 0.013 | <0.001 | <0.001 |
| Stroke | 427 | 0.135 | 0.051 | <0.001 | 0.004 |
| MACE | 2017 | <0.001 | <0.001 | <0.001 | <0.001 |

A P value < 0.05 indicates evidence against the proportional hazards assumption. Models in panel B were adjusted for age, smoking history, BMI, waist circumference, hip circumference, serum creatinine, LDL-C, and total cholesterol.

FFP, Fried Frailty Phenotype; CF, clinical frailty; PF, protein-based frailty; BMI, body mass index; LDL-C, low-density lipoprotein cholesterol; AMI, acute myocardial infarction; CHD, coronary heart disease; HF, heart failure; AF, atrial fibrillation; MACE, major adverse cardiovascular events; PH, proportional hazards.

## Table S7. Time-varying frailty associations in interval-specific Cox sensitivity analyses

### A. Clinical FFP and incident CVD

| **Outcome** | **Frailty** | **<5 years** | **5–<10 years** | **≥10 years** | **Phet** |
| --- | --- | --- | --- | --- | --- |
| Incident CVD | frail | 1.692 (1.561–1.835) | 1.247 (1.116–1.393) | 0.912 (0.767–1.085) | <0.001 |
| Incident CVD | pre-frail | 1.108 (1.062–1.155) | 1.036 (0.988–1.087) | 0.943 (0.885–1.005) | <0.001 |

The heterogeneity P value for FFP was assessed jointly across the two non-reference categories. Therefore, the same P value is shown for both comparisons.

### B. Outcome-specific clinical and protein-based frailty

| **Outcome** | **Frailty** | **<5 years** | **5–<10 years** | **≥10 years** | **Phet** |
| --- | --- | --- | --- | --- | --- |
| AMI | CF | 1.047 (0.782–1.400) | 1.486 (1.107–1.995) | 1.319 (0.890–1.955) | 0.239 |
| AMI | PF | 1.248 (1.064–1.464) | 1.053 (0.879–1.262) | 1.109 (0.874–1.408) | 0.323 |
| CHD | CF | 1.509 (1.328–1.715) | 1.070 (0.914–1.252) | 0.893 (0.709–1.126) | <0.001 |
| CHD | PF | 1.311 (1.223–1.407) | 1.055 (0.961–1.160) | 0.976 (0.849–1.123) | <0.001 |
| HF | CF | 1.579 (1.302–1.916) | 1.222 (0.993–1.503) | 0.819 (0.624–1.076) | <0.001 |
| HF | PF | 1.869 (1.703–2.051) | 1.419 (1.265–1.592) | 1.286 (1.102–1.501) | <0.001 |
| AF | CF | 1.162 (1.025–1.317) | 0.812 (0.693–0.951) | 1.035 (0.837–1.280) | 0.002 |
| AF | PF | 1.214 (1.129–1.305) | 0.993 (0.902–1.092) | 0.865 (0.756–0.991) | <0.001 |
| Stroke | CF | 1.278 (0.962–1.697) | 1.080 (0.833–1.400) | 0.859 (0.618–1.194) | 0.196 |
| Stroke | PF | 1.290 (1.104–1.508) | 1.095 (0.936–1.281) | 0.825 (0.666–1.021) | 0.002 |
| MACE | CF | 1.484 (1.329–1.657) | 1.063 (0.933–1.211) | 0.860 (0.713–1.036) | <0.001 |
| MACE | PF | 1.412 (1.328–1.500) | 1.135 (1.050–1.226) | 0.978 (0.872–1.096) | <0.001 |

Models used the same adjustment covariates and smaller analytic samples as the Schoenfeld residual diagnostics in Table S6 (age, smoking history, BMI, waist and hip circumference, serum creatinine, LDL-C, and total cholesterol). Phet indicates the P value for heterogeneity across follow-up intervals.

FFP, Fried Frailty Phenotype; CF, clinical frailty; PF, protein-based frailty; HR, hazard ratio; CI, confidence interval; AMI, acute myocardial infarction; CHD, coronary heart disease; HF, heart failure; AF, atrial fibrillation; MACE, major adverse cardiovascular events; LDL-C, low-density lipoprotein cholesterol.

## Table S8. Baseline characteristics of the XiDu2017 cohort after imputation (n = 4,323)

| **Variable** | **Robust** | **Pre-frail** | **Frail** |
| --- | --- | --- | --- |
| Participants, n (%) | 1894 (43.8) | 2135 (49.4) | 294 (6.8) |
| Sex, n (%) |  |  |  |
| Male | 885 (46.7) | 826 (38.7) | 84 (28.6) |
| Female | 1009 (53.3) | 1309 (61.3) | 210 (71.4) |
| DM, n (%) |  |  |  |
| Present | 148 (7.8) | 176 (8.2) | 38 (12.9) |
| Absent | 1746 (92.2) | 1959 (91.8) | 256 (87.1) |
| CVD, n (%) |  |  |  |
| Present | 641 (33.8) | 815 (38.2) | 121 (41.2) |
| Absent | 1253 (66.2) | 1320 (61.8) | 173 (58.8) |
| Age, years | 68.46 (6.10) | 70.86 (7.11) | 77.78 (7.54) |
| WHR | 0.90 (0.28) | 0.90 (0.22) | 0.91 (0.26) |
| BMI, kg/m² | 24.15 (3.10) | 23.66 (3.41) | 22.56 (3.56) |
| BUN, mmol/L | 5.76 (1.36) | 6.02 (1.59) | 6.27 (2.23) |
| Scr, μmol/L | 66.47 (13.51) | 66.92 (16.67) | 70.16 (25.63) |
| Hb, g/L | 139.20 (12.96) | 135.35 (13.70) | 129.51 (14.59) |
| UA, μmol/L | 319.48 (81.65) | 320.15 (87.02) | 316.07 (87.69) |
| TC, mmol/L | 5.00 (0.88) | 4.97 (0.89) | 4.94 (0.90) |
| TG, mmol/L | 1.63 (1.08) | 1.58 (1.05) | 1.50 (0.77) |

Values are n (%) or mean (SD). The table summarizes the imputed baseline dataset.

WHR, waist-to-hip ratio; BMI, body mass index; BUN, blood urea nitrogen; Scr, serum creatinine; Hb, hemoglobin; UA, uric acid; TC, total cholesterol; TG, triglycerides; DM, diabetes mellitus; CVD, cardiovascular disease; SD, standard deviation.

## Table S9. Baseline characteristics of the XiDu2017 cohort before imputation (n = 4,323)

| **Variable** | **Robust** | **Pre-frail** | **Frail** |
| --- | --- | --- | --- |
| Participants, n (%) | 1894 (43.8) | 2135 (49.4) | 294 (6.8) |
| Sex, n (%) |  |  |  |
| Male | 885 (46.7) | 826 (38.7) | 84 (28.6) |
| Female | 1009 (53.3) | 1309 (61.3) | 210 (71.4) |
| DM, n (%) |  |  |  |
| Present | 148 (7.8) | 176 (8.2) | 38 (12.9) |
| Absent | 1746 (92.2) | 1959 (91.8) | 256 (87.1) |
| CVD, n (%) |  |  |  |
| Present | 641 (33.8) | 815 (38.2) | 121 (41.2) |
| Absent | 1253 (66.2) | 1320 (61.8) | 173 (58.8) |
| Age, years | 68.54 (6.15) | 70.88 (7.05) | 77.70 (7.62) |
| WHR | 0.90 (0.29) | 0.90 (0.23) | 0.91 (0.28) |
| BMI, kg/m² | 24.14 (3.09) | 23.69 (3.41) | 22.67 (3.50) |
| BUN, mmol/L | 5.75 (1.41) | 6.04 (1.64) | 6.32 (2.36) |
| Scr, μmol/L | 66.44 (13.64) | 67.04 (16.91) | 70.96 (26.89) |
| Hb, g/L | 139.22 (13.16) | 135.48 (13.97) | 128.92 (14.95) |
| UA, μmol/L | 318.55 (82.65) | 319.52 (87.67) | 314.42 (88.92) |
| TC, mmol/L | 5.00 (0.89) | 4.97 (0.91) | 4.92 (0.92) |
| TG, mmol/L | 1.63 (1.12) | 1.58 (1.04) | 1.52 (0.81) |

Values are n (%) or mean (SD). Continuous-variable summaries use the data before imputation.

WHR, waist-to-hip ratio; BMI, body mass index; BUN, blood urea nitrogen; Scr, serum creatinine; Hb, hemoglobin; UA, uric acid; TC, total cholesterol; TG, triglycerides; DM, diabetes mellitus; CVD, cardiovascular disease; SD, standard deviation.

## Table S10. Univariable logistic regression for prevalent CVD in XiDu2017

| **Variable** | **B** | **SE** | **P value** | **OR (95% CI)** |
| --- | --- | --- | --- | --- |
| Sex | -0.091 | 0.064 | 0.155 | 0.913 (0.805–1.035) |
| DM | 0.56 | 0.11 | <0.001 | 1.750 (1.410–2.172) |
| FFP |  |  |  |  |
| Robust |  |  |  |  |
| Pre-frail | 0.188 | 0.066 | 0.004 | 1.207 (1.061–1.373) |
| Frail | 0.313 | 0.128 | 0.015 | 1.367 (1.064–1.757) |
| Age | 0.034 | 0.004 | <0.001 | 1.034 (1.025–1.043) |
| WHR | 0.385 | 0.16 | 0.016 | 1.47 (1.075–2.011) |
| BMI | 0.098 | 0.01 | <0.001 | 1.102 (1.081–1.124) |
| BUN | 0.063 | 0.02 | 0.002 | 1.065 (1.024–1.108) |
| Scr | 0.012 | 0.002 | <0.001 | 1.012 (1.008–1.017) |
| Hb | 0.005 | 0.002 | 0.024 | 1.005 (1.001–1.010) |
| UA | 0.003 | 0 | <0.001 | 1.003 (1.003–1.004) |
| TC | -0.035 | 0.036 | 0.332 | 0.966 (0.901–1.036) |
| TG | 0.15 | 0.032 | <0.001 | 1.161 (1.091–1.236) |

B and SE denote logistic-regression coefficients and standard errors. FFP categories are presented relative to robust participants. Each other variable was evaluated in a separate univariable model.

WHR, waist-to-hip ratio; BMI, body mass index; BUN, blood urea nitrogen; Scr, serum creatinine; Hb, hemoglobin; UA, uric acid; TC, total cholesterol; TG, triglycerides; DM, diabetes mellitus; CVD, cardiovascular disease; FFP, Fried Frailty Phenotype; OR, odds ratio; CI, confidence interval; SE, standard error; B, regression coefficient.

## Table S11. Reported multivariable logistic regression estimates for prevalent CVD in XiDu2017

| **Variable** | **aOR (95% CI)** | **P value** |
| --- | --- | --- |
| UA | 1.002 (1.001–1.003) | <0.001 |
| Scr | 1.006 (1.001–1.010) | 0.016 |
| BMI | 1.098 (1.076–1.120) | <0.001 |
| Age | 1.032 (1.022–1.042) | <0.001 |
| DM | 1.649 (1.319–2.061) | <0.001 |
| FFP | 1.120 (1.004–1.251) | 0.043 |

The model included FFP, age, sex, DM, WHR, BMI, BUN, Scr, Hb, UA, and TG. Only the six statistically significant estimates are shown. FFP was coded 0 (robust), 1 (pre-frail), and 2 (frail); its aOR is per category increase. The maximum VIF was 1.394 and the minimum tolerance was 0.718, both for UA.

WHR, waist-to-hip ratio; BMI, body mass index; BUN, blood urea nitrogen; Scr, serum creatinine; Hb, hemoglobin; UA, uric acid; TG, triglycerides; DM, diabetes mellitus; CVD, cardiovascular disease; FFP, Fried Frailty Phenotype; aOR, adjusted odds ratio; CI, confidence interval; VIF, variance inflation factor.

## Table S12. Fixed-covariate sensitivity analysis of FFP category and prevalent CVD in XiDu2017 (n = 4,323)

| **Variable** | **B** | **SE** | **aOR (95% CI)** | **P value** |
| --- | --- | --- | --- | --- |
| FFP category, per one-level increase | 0.125 | 0.056 | 1.133 (1.015–1.266) | 0.026 |
| Age, per year | 0.035 | 0.005 | 1.035 (1.026–1.045) | <0.001 |
| Female vs male | -0.114 | 0.068 | 0.893 (0.781–1.020) | 0.096 |
| DM, present vs absent | 0.421 | 0.114 | 1.523 (1.219–1.903) | <0.001 |
| BMI, per kg/m² | 0.099 | 0.010 | 1.105 (1.083–1.127) | <0.001 |
| TC, per mmol/L | -0.068 | 0.039 | 0.934 (0.865–1.008) | 0.081 |
| TG, per mmol/L | 0.090 | 0.033 | 1.094 (1.025–1.167) | 0.007 |

Outcome: prevalent CVD (0, absent; 1, present). FFP was coded 0 (robust), 1 (pre-frail), and 2 (frail). Age, sex, DM, BMI, TC, and TG were entered simultaneously, without univariable P-value screening. Continuous covariates were modeled on their original scales. B and SE are logistic-regression coefficients and standard errors; 95% CIs and two-sided P values use Wald methods.

BMI, body mass index; TC, total cholesterol; TG, triglycerides; DM, diabetes mellitus; CVD, cardiovascular disease; FFP, Fried Frailty Phenotype; aOR, adjusted odds ratio; CI, confidence interval; SE, standard error; B, regression coefficient.

## Table S13. Baseline characteristics of eligible XiDu participants included and not included in the longitudinal analysis

| **Variable** | **Included (n=844)** | **Not included (n=1,902)** | **SMD** |
| --- | --- | --- | --- |
| Age, years | 71.02 (7.72) | 69.04 (6.40) | 0.279 |
| Sex, n (%) |  |  | 0.033 |
| Male | 353 (41.8) | 765 (40.2) |  |
| Female | 491 (58.2) | 1137 (59.8) |  |
| FFP category, n (%) |  |  | 0.192 |
| Robust | 342 (40.5) | 911 (47.9) |  |
| Pre-frail | 426 (50.5) | 894 (47.0) |  |
| Frail | 76 (9.0) | 97 (5.1) |  |
| DM, n (%) |  |  | 0.119 |
| Present | 75 (8.9) | 110 (5.8) |  |
| Absent | 769 (91.1) | 1792 (94.2) |  |
| WHR | 0.90 (0.23) | 0.89 (0.21) | 0.045 |
| BMI, kg/m² | 23.36 (3.18) | 23.44 (3.21) | 0.027 |
| BUN, mmol/L | 5.87 (1.44) | 5.87 (1.42) | 0.004 |
| Scr, μmol/L | 66.12 (14.17) | 65.62 (14.98) | 0.034 |
| Hb, g/L | 136.41 (13.68) | 136.23 (13.12) | 0.014 |
| UA, μmol/L | 313.80 (80.23) | 309.81 (81.32) | 0.049 |
| TC, mmol/L | 4.98 (0.87) | 5.00 (0.89) | 0.014 |
| TG, mmol/L | 1.58 (1.10) | 1.53 (1.04) | 0.054 |

Values are n (%) or mean (SD). The comparison is restricted to the 2,746 XiDu2017 participants without baseline CVD: 844 included and 1,902 not included in the longitudinal analysis. SMDs are absolute; the FFP SMD summarizes the three-category distribution.

WHR, waist-to-hip ratio; BMI, body mass index; BUN, blood urea nitrogen; Scr, serum creatinine; Hb, hemoglobin; UA, uric acid; TC, total cholesterol; TG, triglycerides; DM, diabetes mellitus; CVD, cardiovascular disease; SD, standard deviation; FFP, Fried Frailty Phenotype; SMD, standardized mean difference.

## Table S14. Baseline characteristics and follow-up outcomes of the XiDu longitudinal cohort (n = 844)

| **Variable** | **Robust** | **Pre-frail** | **Frail** |
| --- | --- | --- | --- |
| Participants, n (%) | 342 (40.5) | 426 (50.5) | 76 (9.0) |
| Sex, n (%) |  |  |  |
| Male | 160 (46.8) | 164 (38.5) | 29 (38.2) |
| Female | 182 (53.2) | 262 (61.5) | 47 (61.8) |
| DM, n (%) |  |  |  |
| Present | 28 (8.2) | 38 (8.9) | 9 (11.8) |
| Absent | 314 (91.8) | 388 (91.1) | 67 (88.2) |
| Composite endpoint, n (%) |  |  |  |
| Yes | 237 (69.3) | 325 (76.3) | 70 (92.1) |
| No | 105 (30.7) | 101 (23.7) | 6 (7.9) |
| Age, years | 68.63 (6.50) | 71.53 (7.59) | 78.91 (7.73) |
| WHR | 0.89 (0.19) | 0.90 (0.23) | 0.93 (0.36) |
| BMI, kg/m² | 23.64 (2.94) | 23.37 (3.17) | 22.00 (3.82) |
| BUN, mmol/L | 5.66 (1.43) | 5.94 (1.34) | 6.44 (1.84) |
| Scr, μmol/L | 66.09 (12.70) | 65.45 (14.35) | 70.01 (18.42) |
| Hb, g/L | 139.59 (12.47) | 134.99 (13.96) | 130.12 (13.96) |
| UA, μmol/L | 316.34 (77.44) | 312.86 (82.44) | 307.60 (80.66) |
| TC, mmol/L | 5.00 (0.84) | 5.00 (0.89) | 4.86 (0.85) |
| TG, mmol/L | 1.58 (0.94) | 1.61 (1.27) | 1.46 (0.68) |

Values are n (%) or mean (SD). All participants were free of CVD at the 2017 baseline. The follow-up composite endpoint was incident CVD or all-cause death by 2024.

WHR, waist-to-hip ratio; BMI, body mass index; BUN, blood urea nitrogen; Scr, serum creatinine; Hb, hemoglobin; UA, uric acid; TC, total cholesterol; TG, triglycerides; DM, diabetes mellitus; CVD, cardiovascular disease; SD, standard deviation.

## Table S15. Baseline characteristics of the UKB clinical cohort (n = 84,276)

| **Variable** | **Robust** | **Pre-frail** | **Frail** |
| --- | --- | --- | --- |
| Participants, n (%) | 37533 (44.5) | 43649 (51.8) | 3094 (3.7) |
| Ever smoked n (%) |  |  |  |
| No | 14403 (38.6) | 16030 (37.0) | 1126 (36.8) |
| Yes | 22903 (61.4) | 27282 (63.0) | 1932 (63.2) |
| Alcohol status n (%) |  |  |  |
| Current | 34638 (92.4) | 39270 (90.1) | 2456 (79.4) |
| Never | 1738 (4.6) | 2531 (5.8) | 327 (10.6) |
| Prefer not to answer | 32 (0.1) | 78 (0.2) | 15 (0.5) |
| Previous | 1077 (2.9) | 1712 (3.9) | 295 (9.5) |
| DM, n (%) |  |  |  |
| Do not know | 60 (0.2) | 127 (0.3) | 17 (0.5) |
| No | 35607 (95.0) | 39865 (91.5) | 2478 (80.1) |
| Prefer not to answer | 18 (0.0) | 43 (0.1) | 7 (0.2) |
| Yes | 1799 (4.8) | 3554 (8.2) | 591 (19.1) |
| CA n (%) |  |  |  |
| Do not know | 74 (0.2) | 116 (0.3) | 14 (0.5) |
| No | 33067 (88.2) | 37893 (86.9) | 2571 (83.1) |
| Prefer not to answer | 19 (0.1) | 38 (0.1) | 6 (0.2) |
| Yes - you will be asked about this later by an interviewer | 4324 (11.5) | 5542 (12.7) | 502 (16.2) |
| COPD n (%) |  |  |  |
| No | 7355 (97.9) | 8011 (96.8) | 263 (93.3) |
| Yes | 159 (2.1) | 261 (3.2) | 19 (6.7) |
| Walking Pace n (%) |  |  |  |
| Brisk pace | 14368 (38.4) | 11475 (26.5) | 94 (3.1) |
| None of the above | 35 (0.1) | 102 (0.2) | 11 (0.4) |
| Prefer not to answer | 12 (0.0) | 34 (0.1) | 2 (0.1) |
| Slow pace | 0 (0.0) | 5740 (13.2) | 2567 (83.8) |
| Steady average pace | 23020 (61.5) | 25994 (60.0) | 388 (12.7) |
| Tiredness n (%) |  |  |  |
| Do not know | 1520 (4.1) | 1686 (3.9) | 85 (2.7) |
| More than half the days | 0 (0.0) | 2721 (6.2) | 799 (25.8) |
| Nearly every day | 0 (0.0) | 2153 (4.9) | 1112 (36.0) |
| Not at all | 23255 (62.0) | 21533 (49.4) | 373 (12.1) |
| Prefer not to answer | 108 (0.3) | 133 (0.3) | 11 (0.4) |
| Several days | 12602 (33.6) | 15364 (35.2) | 713 (23.1) |
| Depression n (%) |  |  |  |
| Do not know | 1298 (3.5) | 1829 (4.2) | 234 (7.6) |
| More than half the days | 352 (0.9) | 993 (2.3) | 203 (6.6) |
| Nearly every day | 133 (0.4) | 501 (1.1) | 180 (5.8) |
| Not at all | 31541 (84.1) | 34351 (78.8) | 1772 (57.3) |
| Prefer not to answer | 186 (0.5) | 254 (0.6) | 30 (1.0) |
| Several days | 3975 (10.6) | 5662 (13.0) | 674 (21.8) |
| Grip Weak n (%) |  |  |  |
| No | 37533 (100.0) | 31740 (72.7) | 739 (23.9) |
| Yes | 0 (0.0) | 11909 (27.3) | 2355 (76.1) |
| Slow Walking n (%) |  |  |  |
| No | 37388 (100.0) | 37469 (86.7) | 482 (15.8) |
| Yes | 0 (0.0) | 5740 (13.3) | 2567 (84.2) |
| Weight Loss n (%) |  |  |  |
| No | 37533 (100.0) | 43058 (98.6) | 2987 (96.5) |
| Yes | 0 (0.0) | 591 (1.4) | 107 (3.5) |
| Exhaustion n (%) |  |  |  |
| No | 37133 (100.0) | 38195 (88.6) | 1140 (37.1) |
| Yes | 0 (0.0) | 4933 (11.4) | 1931 (62.9) |
| Low Activity n (%) |  |  |  |
| No | 32850 (100.0) | 10796 (25.9) | 235 (7.7) |
| Yes | 0 (0.0) | 30967 (74.1) | 2809 (92.3) |
| Age, years | 66.88 (1.48) | 66.89 (1.48) | 66.96 (1.47) |
| Waist Circumference, cm | 89.6 (12.1) | 93.3 (13.5) | 100.3 (15.5) |
| Hip Circumference, cm | 102.05 (7.59) | 103.89 (9.02) | 107.95 (12.31) |
| Scr, μmol/L | 73.34 (16.64) | 74.46 (20.63) | 77.32 (37.83) |
| Hb, g/dL | 14.24 (1.15) | 14.23 (1.22) | 13.85 (1.37) |
| Urea, mmol/L | 5.77 (1.36) | 5.84 (1.51) | 6.12 (2.14) |
| HDL-C, mmol/L | 1.51 (0.39) | 1.44 (0.38) | 1.35 (0.37) |
| LDL-C, mmol/L | 3.64 (0.88) | 3.55 (0.90) | 3.34 (0.96) |
| Lp(a), nmol/L | 44.43 (48.95) | 44.58 (49.03) | 44.53 (49.22) |
| TG, mmol/L | 1.73 (0.90) | 1.83 (0.97) | 1.98 (1.07) |
| TC, mmol/L | 5.84 (1.16) | 5.69 (1.20) | 5.39 (1.27) |
| Grip Strength, kg | 31.86 (9.86) | 28.73 (10.62) | 20.99 (9.45) |
| SBP, mmHg | 149.41 (19.81) | 148.38 (19.77) | 146.94 (20.67) |
| DBP, mmHg | 82.63 (10.34) | 82.36 (10.52) | 81.64 (11.24) |
| Weight, kg | 75.15 (13.28) | 77.76 (14.95) | 82.40 (18.20) |
| BMI, kg/m² | 26.73 (3.77) | 27.81 (4.55) | 30.35 (6.18) |
| FFP score | 0.00 (0.00) | 1.24 (0.43) | 3.16 (0.37) |

Values are n (%) or mean (SD). Displayed denominators differ across variables. For SI conversion, multiply Hb in g/dL by 10 to obtain g/L and blood pressure in mmHg by 0.133322 to obtain kPa.

BMI, body mass index; Scr, serum creatinine; Hb, hemoglobin; TC, total cholesterol; TG, triglycerides; DM, diabetes mellitus; SD, standard deviation; FFP, Fried Frailty Phenotype; LDL-C, low-density lipoprotein cholesterol; HDL-C, high-density lipoprotein cholesterol; CA, cancer; COPD, chronic obstructive pulmonary disease; Lp(a), lipoprotein(a); SBP, systolic blood pressure; DBP, diastolic blood pressure; UKB, UK Biobank.

## Table S16. Univariable Cox regression for incident CVD in UKB

| **Variable** | **B** | **SE** | **P value** | **HR (95% CI)** |
| --- | --- | --- | --- | --- |
| Age | 0.046 | 0.004 | <0.001 | 1.05 (1.04–1.06) |
| Ever smoked | 0.179 | 0.012 | <0.001 | 1.20 (1.17–1.22) |
| Alcohol status |  |  |  |  |
| Current |  |  |  |  |
| Never | -0.031 | 0.024 | 0.197 | 0.97 (0.92–1.02) |
| Prefer not to answer | 0.184 | 0.127 | 0.149 | 1.20 (0.94–1.54) |
| Previous | 0.220 | 0.026 | <0.001 | 1.25 (1.19–1.31) |
| DM |  |  |  |  |
| Do not know |  |  |  |  |
| No | -0.248 | 0.093 | 0.007 | 0.78 (0.65–0.94) |
| Prefer not to answer | -0.452 | 0.210 | 0.032 | 0.64 (0.42–0.96) |
| Yes | 0.024 | 0.094 | 0.794 | 1.03 (0.85–1.23) |
| BMI | 0.009 | 0.001 | <0.001 | 1.01 (1.01–1.01) |
| Waist Circumference | 0.013 | 0.000 | <0.001 | 1.01 (1.01–1.01) |
| Hip Circumference | 0.006 | 0.001 | <0.001 | 1.01 (1.01–1.01) |
| Scr | 0.004 | 0.000 | <0.001 | 1.00 (1.00–1.00) |
| Hb | 0.035 | 0.005 | <0.001 | 1.04 (1.03–1.04) |
| HDL-C | -0.486 | 0.017 | <0.001 | 0.62 (0.59–0.64) |
| LDL-C | -0.203 | 0.007 | <0.001 | 0.82 (0.81–0.83) |
| TG | 0.008 | 0.006 | 0.18 | 1.01 (1.00–1.02) |
| TC | -0.176 | 0.005 | <0.001 | 0.84 (0.83–0.85) |
| FFP |  |  | <0.001 | 1.32 (1.23–1.39) |
| Frail |  |  |  |  |
| Pre-frail | -0.299 | 0.044 | <0.001 | 0.74 (0.681–0.808) |
| Robust | -0.366 | 0.044 | <0.001 | 0.69 (0.636–0.756) |
| FFP score | 0.093 | 0.011 | <0.001 | 1.09 (1.08–1.11) |

B and SE denote Cox-regression coefficients and standard errors. The FFP score is the number of criteria present (0–5).

BMI, body mass index; Scr, serum creatinine; Hb, hemoglobin; TC, total cholesterol; TG, triglycerides; DM, diabetes mellitus; CVD, cardiovascular disease; FFP, Fried Frailty Phenotype; HR, hazard ratio; CI, confidence interval; SE, standard error; B, regression coefficient; LDL-C, low-density lipoprotein cholesterol; HDL-C, high-density lipoprotein cholesterol; UKB, UK Biobank.

## Table S17. Multivariable Cox regression for incident CVD in UKB

| **Variable** | **aHR (95% CI)** | **P value** |
| --- | --- | --- |
| FFP: frail | 1.380 (1.280–1.480) | <0.001 |
| FFP: pre-frail | 1.050 (1.010–1.080) | 0.005 |
| TC | 0.860 (0.790–0.920) | <0.001 |
| LDL-C | 1.250 (1.140–1.370) | <0.001 |
| Scr | 1.001 (1.001–1.002) | <0.001 |
| Hip circumference | 1.010 (1.000–1.010) | <0.001 |
| Waist circumference | 1.010 (1.010–1.010) | <0.001 |
| BMI | 0.970 (0.970–0.980) | <0.001 |
| Ever smoked | 1.070 (1.030–1.100) | <0.001 |
| Age | 1.050 (1.040–1.060) | <0.001 |

FFP estimates compare frail and pre-frail participants with robust participants. The model adjusted for all covariates listed. Covariates were selected using LASSO regression.

BMI, body mass index; Scr, serum creatinine; TC, total cholesterol; CVD, cardiovascular disease; FFP, Fried Frailty Phenotype; aHR, adjusted hazard ratio; CI, confidence interval; LDL-C, low-density lipoprotein cholesterol; UKB, UK Biobank; LASSO, least absolute shrinkage and selection operator.

## Table S18. Discrimination of CF and PF for cardiovascular outcomes in the full proteomic cohort

| **Outcome** | **Model** | **C-index (95% CI)** | **P value** | **5-year AUC (95% CI)** | **P value** |
| --- | --- | --- | --- | --- | --- |
| AMI | CF | 0.554 (0.524–0.583) |  | 0.534 (0.487–0.580) |  |
| AMI | PF | 0.579 (0.545–0.612) | 0.205 | 0.628 (0.578–0.679) | 0.004 |
| CHD | CF | 0.555 (0.541–0.570) |  | 0.581 (0.560–0.602) |  |
| CHD | PF | 0.614 (0.599–0.630) | <0.001 | 0.652 (0.629–0.675) | <0.001 |
| HF | CF | 0.561 (0.541–0.581) |  | 0.595 (0.563–0.626) |  |
| HF | PF | 0.699 (0.677–0.720) | <0.001 | 0.756 (0.723–0.788) | <0.001 |
| AF | CF | 0.529 (0.515–0.543) |  | 0.547 (0.527–0.568) |  |
| AF | PF | 0.593 (0.577–0.609) | <0.001 | 0.624 (0.600–0.647) | <0.001 |
| Stroke | CF | 0.524 (0.498–0.551) |  | 0.550 (0.503–0.596) |  |
| Stroke | PF | 0.573 (0.543–0.602) | 0.004 | 0.608 (0.559–0.657) | 0.036 |
| MACE | CF | 0.551 (0.542–0.560) |  | 0.583 (0.566–0.599) |  |
| MACE | PF | 0.617 (0.607–0.627) | <0.001 | 0.665 (0.647–0.683) | <0.001 |

Values are estimates (95% CI). P values compare PF with CF for the corresponding metric and outcome. AUC refers to the 5-year time-dependent AUC. The full-cohort results are shown.

CI, confidence interval; CF, clinical frailty; PF, protein-based frailty; AUC, area under the receiver operating characteristic curve; AMI, acute myocardial infarction; CHD, coronary heart disease; HF, heart failure; AF, atrial fibrillation; MACE, major adverse cardiovascular events.

## Table S19. Associations of CF and PF with cardiovascular outcomes in the full proteomic cohort

| **Outcome** | **Frailty measure** | **HR (95% CI)** | **P value** |
| --- | --- | --- | --- |
| AMI | PF | 1.310 (1.176–1.459) | <0.001 |
| AMI | CF | 1.488 (1.218–1.818) | <0.001 |
| CHD | PF | 1.457 (1.386–1.531) | <0.001 |
| CHD | CF | 1.476 (1.339–1.627) | <0.001 |
| HF | PF | 1.989 (1.869–2.116) | <0.001 |
| HF | CF | 1.577 (1.379–1.804) | <0.001 |
| AF | PF | 1.405 (1.336–1.477) | <0.001 |
| AF | CF | 1.236 (1.124–1.360) | <0.001 |
| Stroke | PF | 1.295 (1.175–1.427) | <0.001 |
| Stroke | CF | 1.191 (0.995–1.426) | 0.057 |
| MACE | PF | 1.524 (1.475–1.575) | <0.001 |
| MACE | CF | 1.449 (1.361–1.542) | <0.001 |

Values are HRs (95% CI) and two-sided P values. The full-cohort estimates are shown.

HR, hazard ratio; CI, confidence interval; CF, clinical frailty; PF, protein-based frailty; AMI, acute myocardial infarction; CHD, coronary heart disease; HF, heart failure; AF, atrial fibrillation; MACE, major adverse cardiovascular events.

## Table S20. Incremental discrimination of PF beyond conventional cardiovascular risk factors

### A. C-index

| **Outcome** | **Base model** | **Base + PF** | **ΔC-index** | **P value** |
| --- | --- | --- | --- | --- |
| AMI | 0.642 (0.615–0.677) | 0.644 (0.618–0.679) | 0.002 (−0.002–0.012) | 0.324 |
| CHD | 0.626 (0.613–0.643) | 0.648 (0.633–0.663) | 0.022 (0.014–0.031) | <0.001 |
| HF | 0.634 (0.617–0.658) | 0.716 (0.695–0.736) | 0.082 (0.061–0.101) | <0.001 |
| AF | 0.608 (0.594–0.624) | 0.636 (0.622–0.652) | 0.028 (0.018–0.038) | <0.001 |
| Stroke | 0.602 (0.582–0.635) | 0.612 (0.589–0.644) | 0.010 (0.001–0.022) | 0.004 |
| MACE | 0.603 (0.594–0.613) | 0.643 (0.634–0.654) | 0.040 (0.033–0.048) | <0.001 |

### B. 5-year time-dependent AUC

| **Outcome** | **Base model** | **Base + PF** | **ΔAUC** | **P value** |
| --- | --- | --- | --- | --- |
| AMI | 0.640 (0.597–0.693) | 0.648 (0.606–0.701) | 0.008 (−0.002–0.030) | 0.144 |
| CHD | 0.646 (0.624–0.672) | 0.681 (0.659–0.703) | 0.035 (0.022–0.049) | <0.001 |
| HF | 0.659 (0.630–0.694) | 0.772 (0.743–0.800) | 0.113 (0.077–0.142) | <0.001 |
| AF | 0.628 (0.608–0.650) | 0.670 (0.650–0.689) | 0.042 (0.026–0.057) | <0.001 |
| Stroke | 0.640 (0.591–0.690) | 0.655 (0.607–0.701) | 0.015 (−0.003–0.045) | 0.056 |
| MACE | 0.631 (0.614–0.651) | 0.690 (0.672–0.708) | 0.058 (0.045–0.074) | <0.001 |

Values are bootstrap optimism-corrected estimates (95% CI), based on 500 resamples in the full cohort. The base model included age, sex, smoking status, systolic blood pressure, DM, TC, and HDL-C. Δ denotes Base + PF minus Base.

TC, total cholesterol; DM, diabetes mellitus; CI, confidence interval; HDL-C, high-density lipoprotein cholesterol; PF, protein-based frailty; AUC, area under the receiver operating characteristic curve; AMI, acute myocardial infarction; CHD, coronary heart disease; HF, heart failure; AF, atrial fibrillation; MACE, major adverse cardiovascular events.

## Figure S1. Factors associated with prevalent CVD in XiDu2017

| **XiDu2017** | | | |
| --- | --- | --- | --- |
| **a**   | | **b**   | |
| **c**   | | **d**   | |
| **e**   | **f**   | | **g**   |

(a–b) Associations of prevalent CVD with DM status and FFP category, assessed by chi-square tests.

(c) Time in seconds to complete a 4.57-m walk among participants with and without CVD; this panel reports walking time rather than walking speed.

(d–g) Distributions of weight loss, exhaustion, weakness, and low physical activity by CVD status. XiDu FFP definitions are given in Table S2.

CVD, cardiovascular disease; DM, diabetes mellitus; FFP, Fried Frailty Phenotype.
